# Where we eat is who we are: a survey of food-related travel patterns to Singapore’s hawker centers, food courts and coffee shops

**DOI:** 10.1186/s12966-020-01031-5

**Published:** 2020-10-20

**Authors:** Shin Bin Tan, Mariana Arcaya

**Affiliations:** 1 Massachusetts Institute of Technology, 77 Massachusetts Ave, Cambridge, 02139 MA USA; 2 Lee Kuan Yew School of Public Policy, National University of Singapore, 469C Bukit Timah Rd, Singapore, 259772 Singapore

**Keywords:** Food environments, Food related travel, Travel mode

## Abstract

**Background:**

The development of empirically-grounded policies to change the obesogenic nature of urban environment has been impeded by limited, inconclusive evidence of the link between food environments, dietary behaviors, and health-related outcomes, in part due to inconsistent methods of classifying and analyzing food environments. This study explores how individual and built environment characteristics may be associated with how far and long people travel to food venues,that can serve as a starting point for further policy-oriented research to develop a more nuanced, context-specific delineations of ‘food environments’ in an urban Asian context.

**Methods:**

Five hundred twenty nine diners in eight different neighborhoods in Singapore were surveyed about how far and long they travelled to their meal venues, and by what mode. We then examined how respondents’ food-related travel differed by socioeconomic characteristics, as well as objectively-measured built environment characteristics at travel origin and destination, using linear regression models.

**Results:**

Low-income individuals expended more time traveling to meal destinations than high-income individuals, largely because they utilized slower modes like walking rather than driving. Those travelling from areas with high food outlet density travelled shorter distances and times than those from food-sparse areas, while those seeking meals away from their home and work anchor points had lower thresholds for travel. Respondents also travelled longer distances to food-dense locations, compared to food-sparse locations.

**Conclusion:**

Those seeking to improve food environments of poor individuals should consider studying an intervention radius pegged to typical walking distances, or ways to improve their transport options as a starting point. Policy-focused research on food environments should also be sensitive to locational characteristics, such as food outlet densities and land use.

## Background

Food environments are widely believed to be a driver of increasing obesity rates [1]. Within developed countries such as Australia, Canada, UK, Europe and the US, unhealthy food environments in poorer and minority neighborhoods have also been blamed, in part, for higher obesity rates among poor and minority populations compared to richer, non-minority counterparts [2–4]. However, empirical evidence on how food environments actually affect dietary behaviors and health outcomes such as obesity rates has been inconclusive and limited in scope [5–8].

Despite the difficulty in establishing a clear causal link between food environments and obesity, there is still much interest in policy and planning interventions to change the obesogenic nature of urban environments, particularly for vulnerable groups such as the low-income and children [9–11]. For instance, in 2009, South Korea implemented ‘Green Food Zones’ 200 meters around schools, where sales of unhealthy foods were restricted [12]. In December 2017, the London Mayor announced a similar initiative to ban new fast food outlets within 400m of schools [13].

Such policies that regulate food environments around schools and other locations must designate specific distances in order to operate. Their effectiveness are thus dependent on the accurate identification and definition of the physical extent of one’s food environment and on a proper understanding of how far people are willing to travel for food. However, current literature on food environments does not sufficiently support the accurate identification and definition of a bounded ‘food environment’ because of four key limitations: the inconsistent definitions of ‘food environments’ across studies; a lack of justification for geographic extent and potential spatial misclassification; assumptions of a uniform neighborhood effect across different groups and urban environments; and the exclusion of non-residential environments.

Firstly, food environment boundaries have been inconsistently defined across various research studies. Researchers commonly use administrative units such as census tracts or postal sectors, or buffers of varying radii around address points or centroids of administrative units [6, 14].

Secondly, few food environment studies explicitly justify their choice of buffer distance [6, 15], which may reflect a lack of literature on food-related travel behavior [16] that food environment researchers can draw upon. Furthermore, the few papers on this topic have found that actual trips to restaurants and grocery-stores tended to be longer than commonly assumed buffers in neighborhood environment studies [16–18], which in turn suggests that much of existing neighborhood effects research into ‘food environments’ might be threatened by spatial misclassification, where chosen analytic boundaries fail to capture the true food-related travel behavior[19].

Thirdly, most food environment studies currently assume a uniform neighborhood effect across all groups, despite the fact that the ‘relevant contextual unit’ is likely to vary depending on the population group, location, and the type of built environment [20]. Resolving the ‘uncertain geographic context problem’ [20] in food environment research will thus require studies to account for people’s travel patterns, taste preferences, cost considerations or social/cultural norms [5, 6]. Further motivating the need for deeper exploration into the heterogeneity in food environments’ extents are findings from activity-based travel research, which shows that trip frequencies, trip lengths, mode choice are dependent on both built form and individual socioeconomic characteristics [21–23].

To date, however, few studies explicitly investigate whether the association between food environments and obesity risks might vary by demographic characteristics, such as income or race/ethnicity. Of the empirical studies that have explored the intersection between income, race/ethnicity and environment, these have found that associations between BMI, food environments [24] and neighborhood walkability [3] differed by SES and race/ethnicity. There is also relatively little research on how built environmental characteristics, such as the availability of public transit, might affect one’s food-related travel patterns, and thus the extent of one’s food environment [16, 25]. The few available studies have found some significant associations. For instance, Thornton et al.(2017) tracked 56 participants’ food purchases over two weeks, in Melbourne, Australia, and found that younger age groups tended to travel further for food purchases, as did participants living in richer neighborhoods with lower access to supermarkets [18]. Kerr et al. (2012) found, from a travel survey of 4800 Atlanta residents, that lowest income, non-White participants, those without a degree, and those travelling from less accessible environments travelled further for food [16]. Zenk et al. (2011) found socioeconomic differences in estimated activity spaces of 131 participants in Detroit, U.S: Participants without cars or were not in the labor force had smaller overall activity spaces than counterparts with comparative higher socioeconomic status[26], which in turn suggests that they might have a smaller food environment.

Fourthly, people often move, shop and make food purchases outside of their home environments [8, 27].For instance, Thornton et al.(2017) found many food purchases occur outside participants’ residential neighbourhood [18]. Similarly, a 2010 survey of 50 individuals in Philadelphia found many visited stores beyond traditionally defined residential neighborhoods [28]. However, much of food environment research still focuses on the residential environment. Little attention is paid to food environments around workplaces, schools, and other ‘anchor points’ [5, 7, 27, 29].‘Anchor points’ are places with important material and symbolic meaning for the individuals, around which they organize their daily activities [30]. Transport scholars theorize that one’s use of time and space is conditioned by one’s basic anchor points, such as home, work and school, since the time available for visiting other places for other activities is bounded by departure from and return to these bases. Within these spatio-temporal constraints, Within these spatio-temporal constraints, individuals make locational and scheduling choices to balance time spent on an activity, such as eating a meal, with travel time to a sufficiently attractive option, such as a good restaurant [31, 32]. If people’s food-seeking behaviors differ by anchor points types, then definitions of food environments should then be contingent on the types of land uses, such as residential, entertainment, office, or retail, within these areas. This study thus examines different types of respondent-defined ‘anchor points’, and whether these affect how far and long people travel for food.

To date, most food environment studies have been conducted within the US, Australia, and New Zealand [15, 29], though a growing number of studies have also be carried out in East Asian cities (e.g. [33–38]). There are significant differences between Western and Asian cities in terms of food environment patterns [33, 39], population density and public transport provision [33], as well as social and cultural norms around food. More research focused on Asian cities and how people there interact with their food environments is needed to facilitate context-specific policy formulation within the region. The relative lack of research into food environments in Asia is a particularly pressing concern, given that Asia is home to 54% of the world’s urban population, as of 2018, compared to 7% in North America, 13% in Europe, and 0.7% in Oceania [40]. The region has also seen a rapid increase in Type 2 diabetes and obesity prevalence [41, 42].

This study is based in Singapore, a highly urbanized, densely-populated city-state in South East Asia. While behaviors will necessarily vary by city, there are three reasons why results from Singapore might provide generalizable insights for many other cities in the Asia Pacific region: Singapore has a reputation for being a food paradise where eating outside of the home is a common practice, with about 60% of Singaporeans eating out at least four times a week [43]. The propensity to eat out is shared elsewhere in Asia, in Hong Kong, Taiwan and Malaysia [44]. Secondly, Singapore is a multi-ethnic, multi-religion country with three primary ethnic groups: Chinese, Malay, and Indian. There is thus significant overlap between Singapore’s population composition with its regional neighbors Malaysia, Indonesia, Hong Kong, China, India and others. Thirdly, like many of the major cities in Japan, China, South Korea and South East Asia (e.g. Tokyo, Hongkong, Bangkok, Kuala Lumpur), Singapore has an extensive public transport network, high population density and high built density. Given the similarities in population, food cultures, foodscapes and built form between Singapore and other cities in the region, this study provides a useful case study for researchers, health professionals and planners interested in Asian urban food environments.

This study combines insights from an ecological model of food-related behavior, which asserts that built environment, social, and individual factors interact to affect eating patterns [1, 45] with models of travel behavior which postulate that travel patterns are dependent on built form, locational characteristics, and individual attributes. It examines how individual and built environment characteristics may be associated with how far (distance) and how long (time) people travel to food venues. In doing so, we seek to contribute empirically-informed, theoretically-derived estimates of food environment extents by population group and built environment type, that provides a starting point for further policy-oriented research in Asian cities.

## Data and methods

### Overview of survey

Data for this study was obtained through a short survey at eight locations, administered between 30 Dec 2017 to 20 Jan 2018, and 20 March to 6 May 2019. Sampling was designed to capture a good mix of food travel behaviors, by picking survey locations across a range of food outlet and public transport density locational characteristics. To identify suitable survey locations,we first generated an island-wide population ‘sampling frame’, which comprises a 200mx200m pixel surface for the whole of Singapore, where the height of each part of the surface represents the intensity of the points of interest [46, pg 168]. In this instance, the two points of interest are existing food outlets (restaurants, cafes, food centers), and public transit locations (bus-stops and Mass Rapid Transit (MRT) stations). To generate this surface,we applied a fixed-bandwidth, isotropic Gaussian kernel (sigma=200m) to smooth a geolocated dataset of food outlets, and another dataset of public transit stops. Each pixel cell provides an estimate of the expected number of points per square meter at that location [46, pg 157-158, 168 to 170]. The analysis was carried out in R version 3.4.3, using statistical package ‘spatstat’ version 1.54.

Such kernel density-based spatial analysis techniques which factor in the location of features relative to one another by weighting features closer together more highly have been applied variously to analysis of features such as parks, health care facilities and the food environment [47]. Researchers have recommended using kernel density estimates rather than simple buffer-based methods of estimating food access, as the former is a more precise measure that accounts for relative distances [15, 24, 48].

Pixels were ranked in terms of their food outlet and public transport intensities. Those with both food outlet and public transport intensity scores falling within either the upper and lower ends of their respective intensity spectrum were further classified into four survey target location categories: 1. high densities of food outlets and public transport services; 2. low densities of both; 3. low density of food outlets and high density of public transport; and 4. high density of food outlets and low density of public transport.

Additionally, only locations that had a hawker center, food court or coffeeshop located within were deemed suitable, as the survey was to be administered at these food venues. These food venues offer self-serviced food options sold in a variety of stalls and at lower price points compared to full-service restaurants (See File 1 for photos). These food venues are an intrinsic part of Singapore’s food culture and attract a diverse pool of customers from different socioeconomic, ethnic/race and age groups: A 2018 survey of a representative sample of Singapore residents(n=1103) found 83% of respondents would eat at or buy takeaways from hawker centres at least once a week. Of all possible eating establishments, hawker centers were the most frequented venue in a given month by 35.6% of respondents,then coffeeshops at 35.5%, and food courts at 22.8%. Collectively, these three eating establishment types representive locations where more than 90% of respondents would eat at frequently [49]. It is thus important for policy-makers to understand travel patterns to these types of food venues.

Two sites per category were selected, as follows:

- **Category 1: Good access to public transport and high density of prepared food outlets**: Survey Locations 1 and 2 are both hawker centers in the city center of Singapore, located within mixed-use neighborhoods with high-density residential, retail, office land uses. The first location is Bugis, while the second location is Hong Lim.
- **Category 2: Poor access to public transport, low density of prepared food outlets** : Survey Location 3 is a food court located in the Tanglin neighborhood, a wealthy residential area near a major shopping are. Survey Location 4 is a large coffeeshop located in a largely public housing residential area Jurong West, at the fringe of the Nanyang Technological University.
- **Category 3: Good access to public transport, low density of prepared food outlets**: Location 5 is a food court in Buangkok, a newly developed, high-rise public housing residential neighborhood. Location 6 is a cluster of coffee shops in Bukit Gombak, an older residential area that includes both high-rise public housing and private landed housing. Both survey locations were next to a an MRT station.
- **Category 4: Poor access to public transport and high density of prepared food outlets**: Location 7 is a hawker center in Marine Parade, a high-rise public housing area. Location 8 is a hawker center in Changi Village, an area located at the northern tip of the Singapore island, and which is currently developing into a ‘recreational hub’ with chalets, resorts and hotels nearby.

**Fig. 1:**
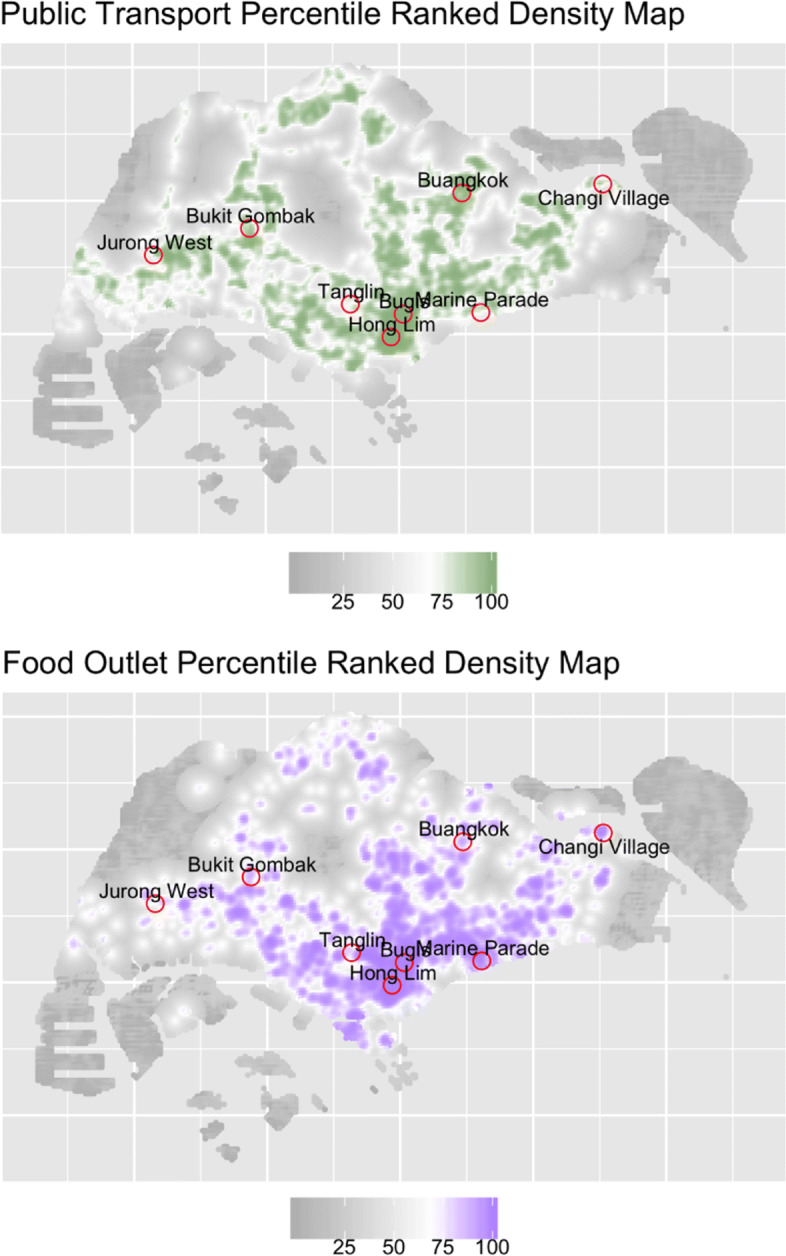
Food Outlet Density and Public Transport Density analysis

Table 1 summarizes the food intensity and public transport intensity estimates at each location. Figure 1 provides a map of the locations, as well as the island-wide intensity maps. Additional File 2 provides an overview of the kernel density estimations and computations for the site selection.

**Table 1:** Food and Public Transport Characteristics of Survey Location

Location	Intensity of Public Transport Services /km2	Intensity of Food Outlets, /km2	Category
Bugis	75	612	Category 1
Hong Lim	66	722	Category 1
Tanglin	7	14	Category 2
Jurong West	10	7	Category 2
Buangkok	27	39	Category 3
Bukit Gombak	19	H28	Category 3
Marine Parade	16	205	Category 4
Changi Village	20	150	Category 4

To recruit participants, the survey utilized purposive sampling at each location. Participants were recruited during one weekend and one weekday per location, during breakfast (8 to 10am), lunch (11.30-1.30pm), and dinner time (5 to 7pm). Surveyers approached individuals or groups who were sitting at the tables either waiting for their food, for other companions to arrive, or who had just finished up their meals. Only individuals who had purchased food were surveyed, to ensure that they were not at the food center for non-food related reasons.

The survey collected information on how far people travelled to their meal venues, how long their trip took, and their mode of travel. Respondents were prompted to indicate either a landmark, building name, postal code or neighborhood from which they departed to arrive at the survey location. Respondents also provided information about their ethnicity, gender, year of birth and household income categories. The survey was administered through an open source Android app ‘ODK-collect’, via a tablet.

### Variables

#### Outcome: travel time and distance

We measure length of travel by both self-reported travel time and calculated Euclidean distance between survey location and self-reported trip origin. Most respondents provided precise origin location markers, such as the exact building or landmark, which enabled precise geocoding. Forty two respondents provided only origin neighborhood, for whom we used the centroid of the administrative planning area (*n*=27)or planning subzone (*n*=15) that best matched their replies as the origin location [50].

#### Origin location characteristics

After geocoding origin locations, we also constructed measures of food outlet density and public transport accessibility around these locations. We extracted the ‘Food Density’ and ‘Public Transport’ intensity scores of 200x200m grid cell centroid closest to their geocoded origin location, which were generated as described in the “Data and methods” section. Self-reported origin types that respondents departed from (e.g. home, school, work) were also included.

#### Travel mode choice

Respondents reported travel mode, which we conceptualize as a potential pathway through which sociodemographic and built environment characteristics influence travel time and cost, from the following response options: ‘car’, ‘public transport’, ‘walking only’ and ‘others’.

#### Individual socio-demographic characteristics

We collected data on socio-demographic variables known to affect food-related travel, such as age [51], gender [27, 52], and income [27]. Household income, which was self-reported by the respondents, was categorized into low (below 2,000 Singapore dollars a month, which falls at approximately 20th percentile nationally), middle (<DOLLAR/>2,000 to <DOLLAR/>10,000, which is up till the 50th percentile), high (<DOLLAR/>10,000 upwards) (Department of Statistics, Singapore 2017). Age was calculated from year of birth reported, and gender response options included ‘Male’, ‘Female’ and an open-ended option.

We also asked respodents to report their race/ethnicity, where response options included: ‘Chinese’, ‘Malay’, ‘Indian’ and an open-ended option, in order to account for the fact that Malay-Muslims observe Halal food restrictions, and may have to travel further to find food that meets their dietary requirements.

### Statistical analysis

We used ordinary least squares linear regressions to estimate associations between individual- and environmental-level characteristics and travel outcomes. We included fixed effects for the eight survey locations, to control for heterogeneity between the different locations, and further clustered standard errors by the survey location, given potential heterogeneity of treatment effects [53], using six-point distribution ‘Wild’ bootstrap method which may improve inference when the number of clusters is small, as is the case here [54]. All models controlled for socio-demographic characteristics such as gender, ethnicity, household income, and age; environmental characteristics of origin and destination points; whether the survey was administered during breakfast, lunch and dinner times; and the types of locations respondents came from.

## Results

**Fig. 2:**
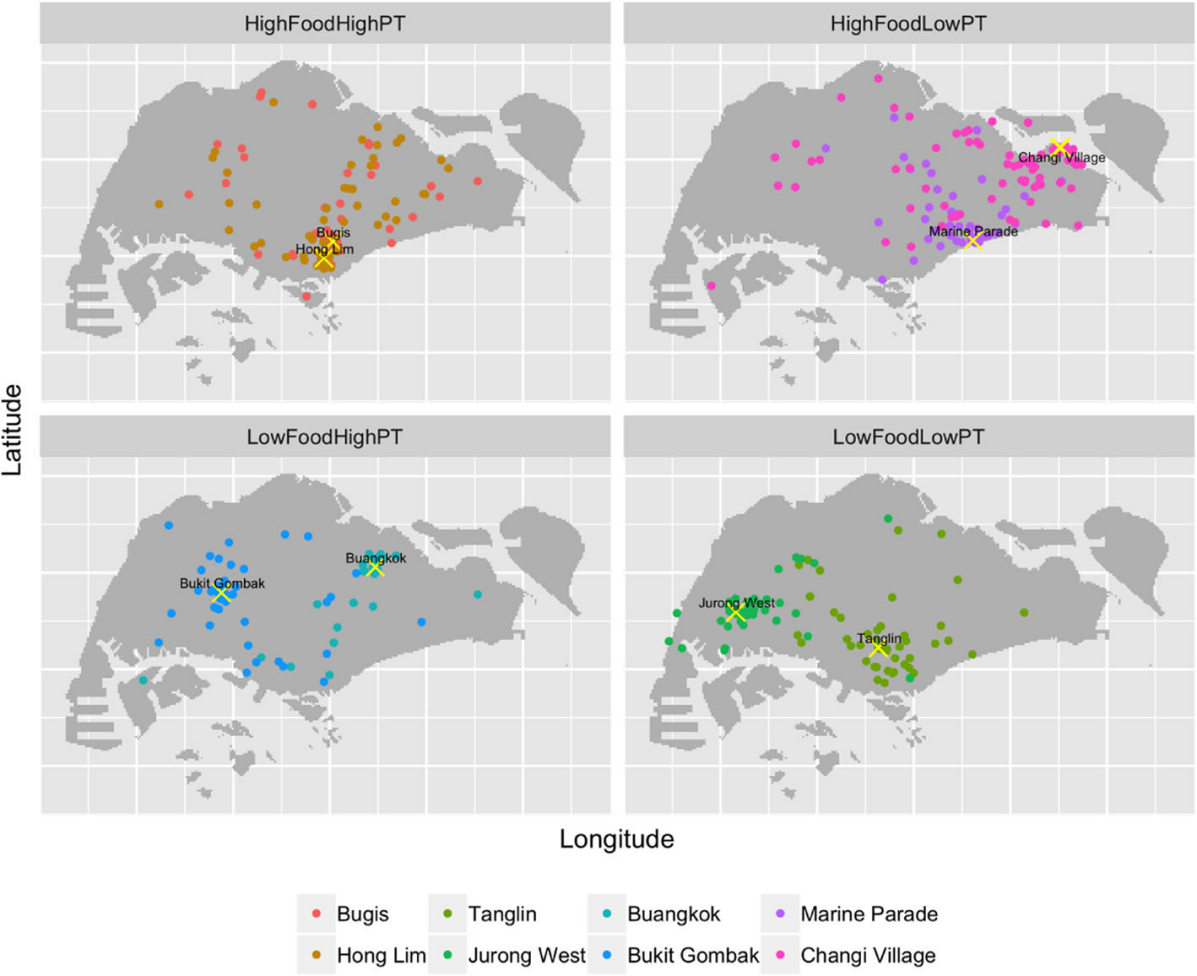
Survey Locations and Where Respondents Originated From

Of 871 people offered a chance to participate in the study, 540 (61%) completed the survey. Of these, 529 included geo-codeable information about the respondent’s origin location. Figure 2 shows the locations of each survey location, and respondents’ origins.

### Respondents’ characteristics

Just over half (57%) of respondents travelled directly from home, about a quarter from work (27%), and about a fifth (16%) from some other place, such as a shopping center, hospital, or exercise venue. Car (38%), walking (31%), and public transport (26%) accounted for nearly all trips, with few respondents (5%) using motorcycle or bicycles. 77% of respondents were of Chinese ethnicity, 7% Malay, 10% Indian and 5% of other ethnicity. No respondents of Malay ethnicity reported household incomes that could be categorized as ‘high’, compared to 19% of Chinese respondents and 11% of Indian respondents who did. This observation is consistent with national statistics which report lower monthly household incomes for Malay-headed households compared to Indian and Chinese headed ones [55]. Table 2 summarizes the characteristics of survey participants.

**Table 2:** Characteristics of surveyed population

Characteristic	N (%)
**Age**	
Mean Years (sd)	41.08 (14.34)
**Ethnicity**	
Chinese	411 (78)
Indian	54 (10)
Malay	36 (7)
Others	28 (5)
**Household Income**	
High Income	85 (16)
Middle Income	285 (54)
Low Income	55 (10)
No Answer	104 (20)
**Travel Mode**	
Car	200 (38)
Public Transport	137 (26)
Walking	164 (31)
Other	28 (5)
**Origin Type**	
Home	301 (57)
Work	142 (27)
Other	86 (16)
**Survey Location**	
Bugis	59 (11)
Hong Lim	71 (13)
Tanglin	56 (11)
Jurong West	66 (12)
Buangkok	54 (10)
Bukit Gombak	83 (16)
Marine Parade	60 (11)
Changi Village	80 (15)

### Average travel distances and times

**Fig. 3:**
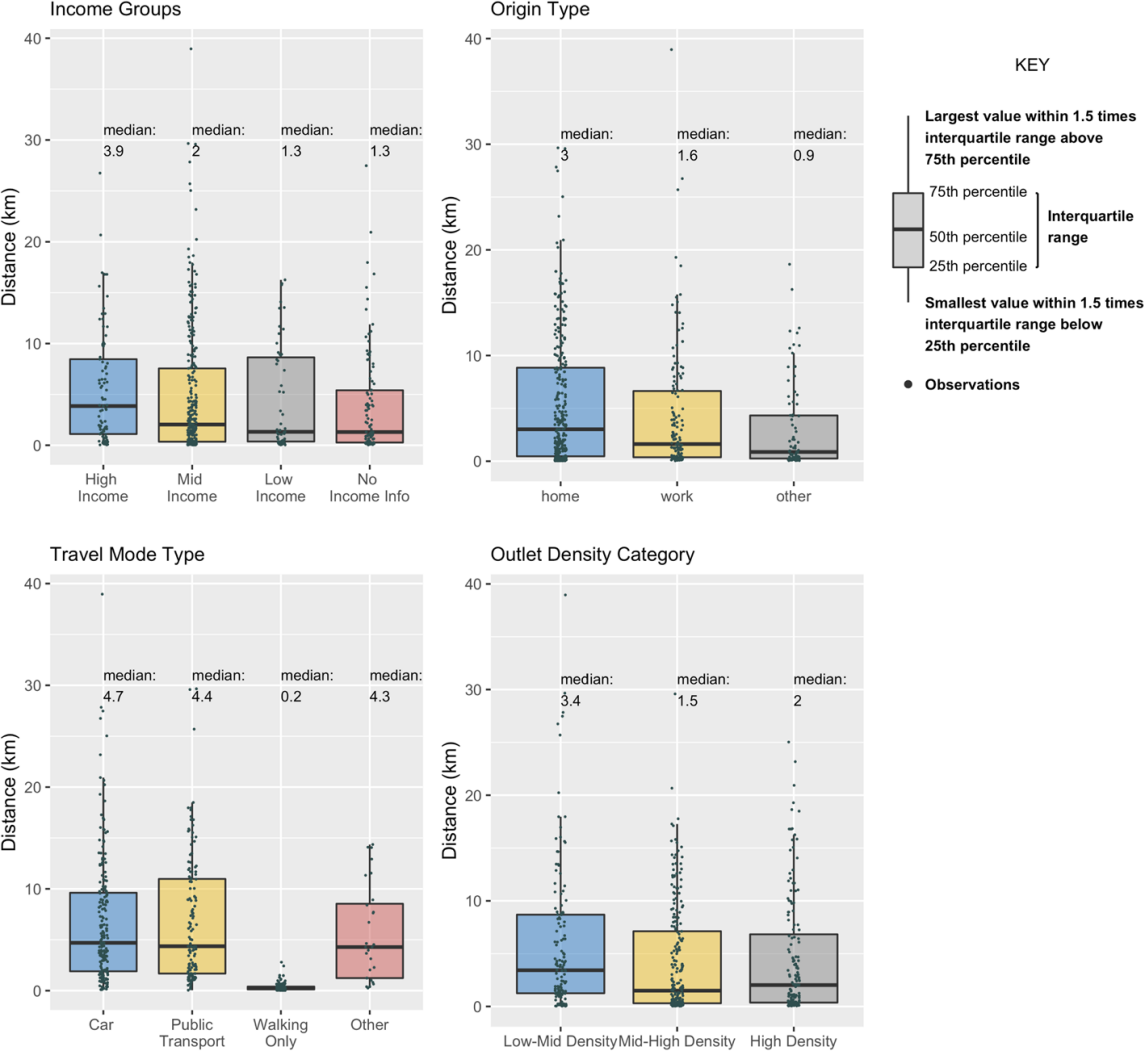
Median travel distances

Respondents travelled a median of 10 min (mean = 17.9, s.d.= 17.7)and 2km (mean=4.7km, s.d.=5.9) to get to their dining destinations. While differences between time and distance travelled on weekdays and weekends were not statistically significant, there were substantial differences in median travel distances by participants’ household income categories, origin types, travel modes and origin food outlet densities (Fig. 3). Here origin food outlet densities were categorized as ‘Low-Mid Density’ for locations at the bottom quartile of all locations in Singapore in terms of food outlet density. Locations at the 25th to 75th percentile, and top quartile were coded ‘Mid-High’ and ‘High Density’ respectively. In contrast, median travel times were largely similar across these participant characteristics except for travel mode, where those travelling by walking only travelled for much shorter times (5 mins) compared to other modes (15mins for car, 25mins for public transport) (See Additional File 3)

### Multivariable models

Age, gender, and ethnicity were not statistically associated with distance or time travelled to food locations but income was negatively associated with travel time (Model i in Tables 3 and 4).

**Table 3:** Income, Origin Built Environment and Distance Travelled

	*Dependent variable:*		
	Travel Distance (km)		
	Model i ^b^	Model ii ^b^	Model iii ^b^
	B (95%CI)	B(95%CI)	B (95%CI)
**Income Category**^**a**^			
Mid Income	−0.65 (−2.39, 1.10)	−0.09 (−1.82, 1.63)	0.19 (−1.58, 1.96)
Low Income	−0.91 (−2.24, 0.43)	0.19 (−0.94, 1.31)	0.56 (−0.60, 1.72)
No Income Info	−1.44 (−3.28, 0.39)	−1.13^∗^ (−2.33, 0.08)	−0.61 (−1.85, 0.63)
**Ethnicity**^**a**^			
Indian	0.41 (−1.64, 2.45)		
Malay	−0.81 (−3.68, 2.07)		
Other Ethnicity	−0.29 (−2.35, 1.77)		
**Age Category**^**a**^			
Under 30	−0.54 (−1.52, 0.44)		
More than 65	−0.54 (−3.67, 2.58)		
Age: no response	−0.53 (−3.13, 2.06)		
**Gender**^**a**^			
Male	0.67 (−0.66, 1.99)		
**Survey Location**^**a**^			
Hong Lim		−0.06 (−0.65, 0.53)	−0.40 (−0.98, 0.18)
Tanglin		−2.72^∗∗∗^ (−4.42,−1.01)	−3.19^∗∗∗^ (−4.38,−1.99)
Jurong West		−5.53^∗∗∗^ (−7.47,−3.59)	−4.21^∗∗∗^ (−5.90,−2.53)
Buangkok		−5.03^∗∗∗^ (−6.27,−3.79)	−3.18^∗∗∗^ (−4.56,−1.80)
Bukit Gombak		−4.93^∗∗∗^ (−6.59,−3.26)	−3.38^∗∗∗^ (−4.85,−1.91)
Marine Parade		−3.14^∗∗∗^ (−4.78,−1.50)	−3.19^∗∗∗^ (−4.22,−2.15)
Changi Village		1.34 (−0.51, 3.19)	0.83 (−0.60, 2.26)
**Origin Type**^**a**^			
Origin: Work		−1.59 (−4.33, 1.16)	−1.23 (−3.75, 1.29)
Origin: Other		−1.05 (−4.15, 2.04)	−0.75 (−3.12, 1.61)
**Time of Survey**^**a**^			
Morning		0.35 (−0.96, 1.66)	0.46 (−0.72, 1.65)
Evening		0.29 (−1.04, 1.61)	0.60^∗^ (−0.15, 1.35)
**Origin’s Food Intensity (km2)**		−0.01^∗∗∗^ (−0.01,−0.002)	−0.002 (−0.01, 0.003)
**Origin’s PT Intensity (km2)**		−0.01 (−0.09, 0.07)	−0.03 (−0.07, 0.01)
**Travel Mode**^**a**^			
Public Transport			−0.07 (−0.96, 0.83)
Walking			−5.06^∗∗∗^ (−6.30,−3.82)
Other			−1.38 (−4.57, 1.80)
**Interactions**			
Work:Morning		2.51 (−1.29, 6.31)	0.66 (−2.82, 4.15)
Other:Morning		−3.11^∗∗∗^ (−5.02,−1.19)	−1.84 (−4.81, 1.13)
Work: Evening		2.00 (−0.99, 5.00)	1.11 (−0.96, 3.18)
Other:Evening		0.97 (−1.67, 3.61)	−0.24 (−2.36, 1.89)
**Constant**	5.29^∗∗∗^ (3.52, 7.05)	8.61^∗∗∗^ (4.50, 12.73)	9.37^∗∗∗^ (6.46, 12.28)
Observations	529	529	529
R^2^	0.01	0.24	0.36
Adjusted R^2^	-0.004	0.21	0.33

^∗^*p*<0.1; ^∗∗^*p*<0.05; ^∗∗∗^*p*<0.01

^a^Reference Categories for Categorical Variables: High Income,Chinese, Age 30-65, Female, Bugis, Home, Afternoon, Car

^b^Regression coefficients(B) with (95 confidence interval) are from ordinary least squares linear regression models, with survey location modeled as a fixed effect

**Table 4:** Income, Origin Built Environment and Time (mins) Travelled

	*Dependent variable:*		
	Travel Time (mins)		
	Model i ^b^	Model ii ^b^	Model iii ^b^
	B (95%CI)	B(95%CI)	B (95%CI)
**Income Category**^**a**^			
Mid Income	1.06 (−3.96, 6.08)	2.66 (−3.25, 8.57)	0.35 (−3.82, 4.52)
Low Income	5.00^∗∗^ (0.34, 9.66)	8.07^∗∗^ (1.70, 14.43)	4.36^∗^ (−0.14, 8.87)
No Income Info	1.74 (−2.44, 5.92)	2.77 (−1.03, 6.56)	1.62 (−2.01, 5.25)
**Ethnicity**^**a**^			
Indian	0.17 (−5.81, 6.15)		
Malay	0.42 (−9.29, 10.12)		
Other Ethnicity	−2.84 (−8.95, 3.28)		
**Age Category**^**a**^			
Under 30	−0.29 (−4.43, 3.84)		
More than 65	−1.04 (−7.80, 5.71)		
Age: no response	-4.51 (-12.61, 3.59)		
**Gender**^**a**^			
Male	1.32 (−1.72, 4.35)		
**Survey Location**^**a**^			
Hong Lim		1.60 (−0.60, 3.79)	1.72^∗∗^ (0.10, 3.34)
Tanglin		−10.19^∗∗∗^ (−14.42,−5.97)	−5.87^∗∗^ (−11.15,−0.59)
Jurong West		−15.00^∗∗∗^ (−19.80,−10.20)	−6.43^∗∗^ (−12.45,−0.41)
Buangkok		−15.93^∗∗∗^ (−19.12,−12.74)	−9.07^∗∗∗^ (−12.78,−5.36)
Bukit Gombak		−11.34^∗∗∗^ (−15.52,−7.17)	−3.66 (−8.63, 1.32)
Marine Parade		−4.57^∗∗^ (−8.67,−0.47)	−2.51 (−6.19, 1.16)
Changi Village		−0.78 (−5.72, 4.16)	2.69 (−3.06, 8.45)
**Origin Type**^**a**^			
Origin: Work		−3.72 (−9.65, 2.21)	−2.10 (−7.10, 2.89)
Origin: Other		1.99 (−6.84, 10.83)	2.51 (−4.10, 9.12)
**Time of Survey**^**a**^			
Morning		1.58 (−2.07, 5.24)	1.93^∗^ (−0.40, 4.26)
Evening		1.32 (−2.86, 5.50)	2.15 (−0.76, 5.05)
**Origin’s Food Intensity (km2)**		−0.01^∗∗^ (−0.03,−0.001)	−0.001 (−0.01, 0.01)
**Origin’s PT Intensity (km2)**		−0.07 (−0.29, 0.15)	−0.11 (−0.25, 0.04)
**Travel Mode**^**a**^			
Public Transport			12.09^∗∗∗^ (4.92, 19.27)
Walking			−9.84^∗∗∗^ (−13.09,−6.60)
Other			6.83^∗∗^ (0.50, 13.16)
**Interactions**			
Work:Morning		14.04 (−4.00, 32.07)	4.76 (−11.75, 21.27)
Other:Morning		−12.66^∗∗∗^ (−16.84,−8.47)	−7.47^∗∗^ (−13.62,−1.33)
Work: Evening		7.76 (−2.88, 18.40)	3.60 (−2.65, 9.85)
Other:Evening		−0.80 (−7.00, 5.41)	−5.17 (−12.61, 2.27)
**Constant**	16.12^∗∗∗^ (11.99, 20.26)	24.54^∗∗∗^ (15.92, 33.17)	20.91^∗∗∗^ (12.34, 29.49)
Observations	527	527	527
R^2^	0.01	0.17	0.35
Adjusted R^2^	-0.01	0.14	0.32

^∗^*p*<0.1; ^∗∗^*p*<0.05; ^∗∗∗^*p*<0.01

^a^Reference Categories for Categorical Variables: High Income,Chinese, Age 30-65, Female, Bugis, Home, Afternoon, Car

^b^Regression coefficients(B) with (95 confidence interval) are from ordinary least squares linear regression models, with survey location modeled as a fixed effect

On average, low-income individuals took a significantly longer time to travel to their food venues, compared to high-income individuals, even after controlling for mode of travel. However, while they travelled for shorter distances on average, this association was weak to non-significant. Those who did not provide income information travelled 1.66km less than high income individuals, but did not take a significantly different amount of time doing so. Adding the travel mode variables attenuated this marginally significant association to non-significance. This suggests that the observed relationship between travel distance and income is largely attributable to income-specific differences in mode choice.

The origin type (e.g. home, work, other) and characteristics of origin distance mattered little in relation to travel time and distance, with the exception of travelling from non-work, non-home locations during morning hours. Those who travelled from ‘other’ locations for breakfast travelled 7.5 min less than the base group, even after factoring in travel mode choices (Table 4, Model iii)

Travelling from locations with higher food outlet intensity was associated with shorter travel distances and times (Model ii in Tables 3 & 4). This relationship attenuates to insignificance once travel mode was included, again suggesting that this difference is largely accounted for by “Travel mode choice”. Those travelling from high-density food areas were more likely to walk to nearby food options compared to those in food-sparse areas who more likely travelled by car or public transport. In contrast, the relative densities of public transport services at respondents’ origins were not significantly associated with reported travel time or distance.

Those surveyed at food-rich locations Hong Lim and Changi Village travelled for similar amounts of time and distance as those visiting food-rich Bugis (Tables 3 and 4). In contrast, those surveyed at food-sparse locations travelled for substantially less than those travelling to food-rich Bugis (-11 to -16 min, Table 4, Model B1.ii). After controlling for travel mode however, the time difference attenuates substantially (Table 4, Model iii). Differences in mode choices thus account for some of the observed travel time differences. Regardless of whether the models controlled for travel mode, the modelled results suggest that individuals who ate at the food-sparse locations (Tanglin, Jurong West, Buangkok, Bukit Gombak) travelled about 3 to 5km less, compared to those surveyed at food-rich Bugis.

One exception to the above observed relationship between food outlet intensity at destination and travel cost is Marine Parade. Those travelling to food-rich Marine Parade also travelled, on average, about 3km less than those travelling to Bugis. One possible explanation is that getting to the Marine Parade location is more time-consuming because of the particularly poor transport connectivity, compared to an area like Bugis with excellent road and public transport connectivity. Thus even though respondents spent roughly similar amounts of time traveling to the Marine Parade location compared to Bugis, the former group covered significantly less distance within the same amount of time. In contrast, because the public transport accessibility at Changi Village is better compared to Marine Parade (see Table 1) respondents may not have been as bogged down by slow travel.

While there was no significant difference in travel distances between those travelling by public transport and those by car, the former travelled on average 12.1 min more than the latter. In contrast, those who walked travelled for shorter periods and distances (-9.8 min, -5.1 km) This makes intuitive sense, since walking is both the most physically taxing and slowest mode of travel. Those who walk would thus likely have a lower threshold for time spent travelling, which naturally also restricts the distance one can cover. Of those who travelled by other modes, they also took a longer time on average to get to their meal venues but did not travel significantly longer distances.

## Discussion

The four key findings from our study are as follows: 1) Low-income individuals expended more time traveling to hawker centers, food courts or coffee shops, than high-income individuals, largely because they utilized slower modes like walking rather than driving. 2) Those travelling from areas with high food outlet density travelled shorter distances and times than those from food-sparse areas,in part because they chose to walk to their meal venues. 3) Generally, the type of origin (e.g. home, work etc.) that respondents were travelling from, as well as meal time, was not associated with any significant differences in travel time or distance, with one exception: those who travelled from locations other than home or work travelled shorter distances and time durations to their food venues than their counterparts. 4) Respondents also travelled longer distances to food-dense locations, compared to food-sparse locations

When interpreting the results, one might argue that it is artificial to analyze travel distance and travel cost while controlling for travel mode because the choice of eating location and choice of mode are interdependent, simultaneous decisions. For instance, a person who wishes to eat near to his/her original location is also more likely to choose to walk, whereas someone who wishes to travel farther might drive or take public transport instead. In this study’s interpretation of the findings, results from models that included travel model as well as those from models that did not are examined in parallel, bearing in mind the interdependence between mode choice, travel distance and travel time.

Finding 1: Our findings suggests that low income individuals tended to travel by slower modes such as walking, and thus took a longer time to access the same food options as their richer counterparts. Our finding thus suggests that those seeking to improve the food environments of poor individuals should thus consider studying an intervention radius pegged to typical walking distances of this group. As a reference point, walkers with low household income in our sample (n=77) walked a median distance of 310m, which is shorter than the typical 400m benchmark used in many studies. Furthermore, those analyzing ways to improve food access of the less well-off should also specifically consider how to enhance transport access, which would help reduce the time burden of travelling for food.

Finding 2:The finding that those travelling from more food-dense areas tended to travel for shorter distances and times is intuitive, as trip lengths are generally shorter at locations that have higher destination accessibility [21]. Thus, if food accessibility in one’s originating neighborhood is high, one would have less need to travel far out compared to others travelling from a food-poor area. Interventions to improve food options in food-dense areas should thus also consider studying a smaller radius of focused implementation for the most impact. As highlighted in Fig. 3, those who travelled from origin locations with high food density travelled a median distance of 2km, compared to those travelling from low food density areas who travelled a median distance of 3.4km.

Finding 3: Our findings here also that individuals seeking meals away from their home and work anchor points have lower thresholds for food-related travel. Thus policy-makers considering the radius of intervention in areas such as shopping districts, entertainment venues, or sports and recreational areas might consider a narrower radius than in predominantly residential or work areas. As highlighted in Fig. 3, survey participants traveling from home traveled a median distance of 3.0km, while those leaving from non-work, non-home locations had a median travel distance of 0.9km.

Finding 4: In terms of differences by destination characteristics, our findings suggest that people may be more willing to travel longer distances to food-dense locations, compared to food-sparse locations. Locations with more food options may potentially act as relative ‘attractors’, compared to the food-sparse neighborhoods. Since the effective reach of food-dense neighborhoods seems larger, those interested in improving the ‘healthfulness’ of food environments may consider prioritizing efforts in food-dense neighborhoods.

### Limitations and recommendations for further study

Our study was based on surveys conducted at self-service hawker centers, food courts and coffee shops. While these types of eating venues are very popular in Singapore, our findings nonetheless do not capture travel patterns to the full spectrum of eating venue types, such as full service restaurants or fast food restaurants. More research is thus needed to understand travel behaviors to a wider range of food venues

Given the relatively lower price points of foods offered at self-service hawker centers, coffee shops, and food courts, there are likely to be fewer wealthy individuals there than in higher-end, full service restaurants. Our findings here may thus not reflect the food travel patterns of the very wealthy. Similarly, our findings may not reflect the travel patterns of the very poor who might not be able to afford eating out at all. Our findings might not thus be generalizable to those at the margins of Singapore’s income distribution.

Another potential exclusion bias is that those willing to take the survey may have more flexibility in their schedules, and may also have a higher travel time threshold. The survey findings may thus not represent people with limited time resources. Findings from the survey may thus represent an ‘upper-bound’ in terms of travel times and distances.

Furthermore, administering the survey at food outlets meant this study captured the individuals who ventured out to eat, as those who relied heavily on home-cooked food or who faced mobility challenges would not be at the food outlets to be surveyed. The study findings cannot thus be generalized to this group of individuals. Additional studies on individuals who seldom eat out would be necessary to fill the data gap here.

The study also relies on web-scraped food listings, in additional to government data for hawker center food, as the primary data source for where restaurants and other food outlets are located, as well as the type of food is offered in each restaurant. Food listing websites however may have a particular bias in terms of picking up restaurants and eateries that have a customer base that includes those who are technologically savvy. Eateries whose customer base consists mainly of older, less tech-savvy population may thus not be reflected in the listings. Nevertheless, given the high penetration of smartphones, internet access and social media use in Singapore (e.g. an 2017 report estimated that 95% of Singaporeans have a smartphone and 81% have home internet access),we hypothesize that this may be a fairly small subset of restaurants.

As this study included only eight survey locations, analyses about the relationship between food destinations and food-related travel behavior are at best tentative. Additional research to include a wider selection of survey destinations across different built environment characteristics would be necessary to verify these initial observations.

Finally, this paper presents early explorations of associations between individual, built environment characteristics and food-related travel behavior. More qualitative and quantitative research to understand why and how individuals make food-related travel choices will be needed to build up a strong empirical base to guide policy-makers.

## Conclusion

This study contributes to existing research on food environments, by providing insights about how people travel to meals at popular, self-service eating venues in a high-density Asian city, a relatively understudied urban context. Additionally, this study also provided interesting insights into whether and how people’s food-travel behaviors differed by the type location they were travelling from, beyond the usual focus on residential neighborhoods. Empirical findings from this exploratory study provide policy-makers, urban planners and researchers with a starting point for further policy-oriented research to develop a more nuanced, context-specific delineations of ‘food environments’.

## Supplementary information

**Additional file 1** photos of hawker centers, food courts in Singapore

**Additional file 2** kernel density estimations and site selection

**Additional file 3** median travel times

## Data Availability

The datasets generated during and/or analysed during the current study available from the corresponding author on reasonable request.

## References

[1] Glanz K, Sallis J, Saelens B, Frank L (2005). Healthy nutrition environments: concepts and measures. Am J Health Promot.

[2] Cummins S, Macintyre S (2006). Food environments and obesity-neighbourhood or nation?. International Journal of Epidemiology.

[3] Lovasi G, Hutson M, Guerra M, Neckerman K (2009). Built Environments and Obesity in Disadvantaged Populations. Epidemiol Rev.

[4] Raja S, Yadav P, Changxing Ma (2008). Beyond Food Deserts: Measuring and Mapping Racial Disparities in Neighborhood Food Environments. J Plan Educ Res.

[5] Lytle L, Sokol R (2017). Measures of the food environment: A systematic review of the field, 2007–2015. Health Place.

[6] Gamba R, Schuchter J, Rutt C, Seto E (2015). Measuring the Food Environment and its Effects on Obesity in the United States: A Systematic Review of Methods and Results. J Community Health.

[7] Cobb L, Appel L, Franco M, Jones-Smith J, Nur A, Anderson C (2015). The relationship of the local food environment with obesity: A systematic review of methods, study quality, and results. Obesity.

[8] Block J, Seward M, James P. Neighborhoods and Health In: Duncan D, Kawachi I, editors.. Oxford University Press: 2018. Google-Books-ID: NrNSDwAAQBAJ.

[9] Cheadle A, Samuels S, Rauzon S, Yoshida S, Schwartz P, Boyle M, Beery W, Craypo L, Solomon L (2010). Approaches to Measuring the Extent and Impact of Environmental Change in Three California Community-Level Obesity Prevention Initiatives. Am J Public Health.

[10] Cummins S (2005). Large scale food retailing as an intervention for diet and health: quasi-experimental evaluation of a natural experiment. J Epidemiol Community Health.

[11] Ortega A, Albert S, Chan-Golston A, Langellier B, Glik D, Belin T, Garcia R, Brookmeyer R, Sharif M, Prelip M. Substantial improvements not seen in health behaviors following corner store conversions in two Latino food swamps. BMC Public Health. 2016; 16. 10.1186/s12889-016-3074-1. Accessed 04 Nov 2017. 10.1186/s12889-016-3074-1 PMC4864998 27169514

[12] Bae S, Kim J, Kim K, Park S, Bae J, Lee W (2012). Changes in Dietary Behavior Among Adolescents and Their Association With Government Nutrition Policies in Korea, 2005-2009. J Prev Med Public Health.

[13] Rustin S. Fast food nation: do more takeaways near schools affect younger pupils’ diets?The Guardian. 2017. http://www.theguardian.com/inequality/2017/dec/01. fast-food-nation-do-more-takeaways-near-schools-affect-younger-pupils-diet. Accessed 9 Apr 2018.

[14] Diez Roux A (2016). Neighborhoods and Health: What Do We Know? What Should We Do?. Am J Public Health.

[15] Caspi C, Sorensen G, Subramanian S, Kawachi I (2012). The local food environment and diet: A systematic review. Health & Place.

[16] Kerr J, Frank L, Sallis J, Saelens B, Glanz K, Chapman J (2012). Predictors of trips to food destinations. Int J Behav Nutr Phys Act.

[17] Liu J, Han B, Cohen D. Beyond Neighborhood Food Environments: Distance Traveled to Food Establishments in 5 US Cities, 2009-2011. Prev Chronic Dis. 2015; 12. 10.5888/pcd12.150065. Accessed 01 Nov 2019. 10.5888/pcd12.150065 PMC4552139 26247426

[18] Thornton L, Crawford D, Lamb K, Ball K (2017). Where do people purchase food? A novel approach to investigating food purchasing locations. Int J Health Geogr.

[19] Duncan D, Kawachi I, Subramanian S, Aldstadt J, Melly S, Williams D (2014). Examination of How Neighborhood Definition Influences Measurements of Youths’ Access to Tobacco Retailers: A Methodological Note on Spatial Misclassification. Am J Epidemiol.

[20] Kwan M-P (2012). The Uncertain Geographic Context Problem. Ann Assoc Am Geogr.

[21] Ewing R, Cervero R (2010). Travel and the Built Environment. J Am Plan Assoc.

[22] Bhat C, Koppelman F (1999). A retrospective and prospective survey of time-use research. Transportation; New York.

[23] Rasouli S, Timmermans H (2014). Activity-based models of travel demand: promises, progress and prospects. Int J Urban Sci.

[24] Jones-Smith J, Karter A, Warton E, Kelly M, Kersten E, Moffet H, Adler N, Schillinger D, Laraia B (2013). Obesity and the Food Environment: Income and Ethnicity Differences Among People With Diabetes: The Diabetes Study of Northern California (DISTANCE). Diabetes Care.

[25] Jiao J, Moudon A, Drewnowski A (2011). Grocery Shopping How Individuals and Built Environments Influence Choice of Travel Mode. Transp Res Rec.

[26] Zenk S, Schulz A, Matthews S, Odoms-Young A, Wilbur J, Wegrzyn L, Gibbs K, Braunschweig C, Stokes C (2011). Activity Space Environment and Dietary and Physical Activity Behaviors: A Pilot Study. Health & place.

[27] Li J, Kim C. Measuring Individuals’ Spatial Access to Healthy Foods by Incorporating Mobility, Time, and Mode: Activity Space Measures. Prof Geogr. 2017:1–11. 10.1080/00330124.2017.1338591. Accessed 15 Nov 2017.

[28] Hirsch J, Hillier A (2013). Exploring the Role of the Food Environment on Food Shopping Patterns in Philadelphia, PA, USA: A Semiquantitative Comparison of Two Matched Neighborhood Groups. Int J Environ Res Public Health.

[29] Kelly B, Flood V, Yeatman H (2011). Measuring local food environments: An overview of available methods and measures. Health & Place.

[30] Chaix B, Méline J, Duncan S, Merrien C, Karusisi N, Perchoux C, Lewin A, Labadi K, Kestens Y (2013). GPS tracking in neighborhood and health studies: A step forward for environmental exposure assessment, a step backward for causal inference?. Health & Place.

[31] Dijst M, Vidakovic V (2000). Travel time ratio: the key factor of spatial reach. Transportation.

[32] Susilo Y, Dijst M (2010). Behavioural decisions of travel-time ratios for work, maintenance and leisure activities in the Netherlands. Transp Plan Technol.

[33] Kim D, Lee C, Seo D (2016). Food deserts in Korea? A GIS analysis of food consumption patterns at sub-district level in Seoul using the KNHANES 2008-2012 data. Nutr Res Pract.

[34] Park S, Choi B, Wang Y, Colantuoni E, Gittelsohn J (2013). School and Neighborhood Nutrition Environment and Their Association With Students’ Nutrition Behaviors and Weight Status in Seoul, South Korea. J Adolesc Health.

[35] Choi Y, Suzuki T (2013). Food deserts, activity patterns, & social exclusion: The case of Tokyo, Japan. Applied Geography.

[36] Hanibuchi T, Kondo K, Nakaya T, Nakade M, Ojima T, Hirai H, Kawachi I (2011). Neighborhood food environment and body mass index among Japanese older adults: results from the Aichi Gerontological Evaluation Study (AGES). Int J Health Geogr.

[37] Su S, Li Z, Xu M, Cai Z, Weng M (2017). A geo-big data approach to intra-urban food deserts: Transit-varying accessibility, social inequalities, and implications for urban planning. Habitat International.

[38] Qiujun Wei, Jiangfeng She, Shuhua Zhang, Jinsong Ma (2018). Using Individual GPS Trajectories to Explore Foodscape Exposure: A Case Study in Beijing Metropolitan Area. Int J Environ Res Public Health.

[39] Caspi C, Lenk K, Pelletier J, Barnes T, Harnack L, Erickson D, Laska M. Association between store food environment and customer purchases in small grocery stores, gas-marts, pharmacies and dollar stores. Int J Behav Nutr Phys Act. 2017; 14. 10.1186/s12966-017-0531-x. Accessed 09 Nov 2017. 10.1186/s12966-017-0531-x PMC5460502 28583131

[40] United Nations DepartmentofEconomicandSocialAffairs (2019). World Urbanization Prospects: The 2018 Revision (ST/ESA/SER.A/420).

[41] Ramachandran A, Snehalatha C (2010). Rising Burden of Obesity in Asia. J Obesity.

[42] Yoon K-H, Lee J-H, Kim J-W, Cho J, Choi Y-H, Ko S-H, Zimmet P, Son H-Y (2006). Epidemic obesity and type 2 diabetes in Asia. The Lancet.

[43] Board H. National Nutrition Survey, 2010, Singapore. Technical report HPB. 2013.

[44] Nielsen. What’s In Our Food And On Our Mind - Ingredient And Dining-Out Trends Around The World: The Nielsen Company; 2016.

[45] Sallis J, Owen N, Fisher E. Ecological Models of Health Behavior: John Wiley & Sons; 2008, pp. 467–85. https://psycnet.apa.org/record/2008-17146-020.

[46] Baddeley A, Rubak E, Turner R. Spatial Point Patterns : Methodology and Applications with R,; 2016. https://lib.mit.edu/record/cat00916a/mit.002433733. Accessed 30 July 2020.

[47] King T, Bentley R, Thornton L, Kavanagh A. Using kernel density estimation to understand the influence of neighbourhood destinations on BMI. BMJ Open. 2016; 6(2). 10.1136/bmjopen-2015-008878. Accessed 29 Nov 2017. 10.1136/bmjopen-2015-008878 PMC4762106 26883235

[48] Morgan Hughey S, Kaczynski A, Porter D, Hibbert J, Turner-McGrievy G, Liu J (2019). Development and testing of a multicomponent obesogenic built environment measure for youth using kernel density estimations. Health & Place.

[49] Agency N. High majority of Patrons Satisfied with Hawker Centres. Library Catalog: www.nea.gov.sg. 2019. https://www.nea.gov.sg/media/news/news/index/high-majority-of-patrons-satisfied-with-hawker-centres. Accessed 31 July 2020.

[50] Authority U. Master Plan 2014 Subzone Boundary (No Sea). Library Catalog: data.gov.sg.https://data.gov.sg/dataset/master-plan-2014-subzone-boundary-no-sea. Accessed 12 Apr 2020.

[51] Cao X, Mokhtarian P, Handy S (2009). The relationship between the built environment and nonwork travel: A case study of Northern California. Transp Res A Policy Pract.

[52] Scheiner J (2014). Gendered key events in the life course: effects on changes in travel mode choice over time. J Transp Geogr.

[53] Abadie A, Athey S, Imbens GW, Wooldridge J. When should you adjust standard errors for clustering?. No. w24003. National Bureau of Economic Research. 2017. https://www.nber.org/papers/w24003.

[54] Webb M. Reworking wild bootstrap based inference for clustered errors. No. 1315. Queen’s Economics Department Working Paper. 2013. http://hdl.handle.net/10419/97480.

[55] Statistics Do. General Household Survey 2015 Technical report. Singapore: Department of Statistics, Ministry of Trade and Industry.

