# Supplementary material for: Where we eat is who we are: a survey of food-related travel patterns to Singapore’s hawker centers, food courts and coffee shops
Source: Int J Behav Nutr Phys Act. 2020 Oct 20;17:132. doi: 10.1186/s12966-020-01031-5 (PMC7574174; doi:10.1186/s12966-020-01031-5)
Supplement: Supplementary file 1 — Additional file 1 photos of hawker centers, food courts in Singapore [file 12966_2020_1031_MOESM1_ESM.docx]

**Additional File 1: Photos of Hawker Centers and Food Courts**


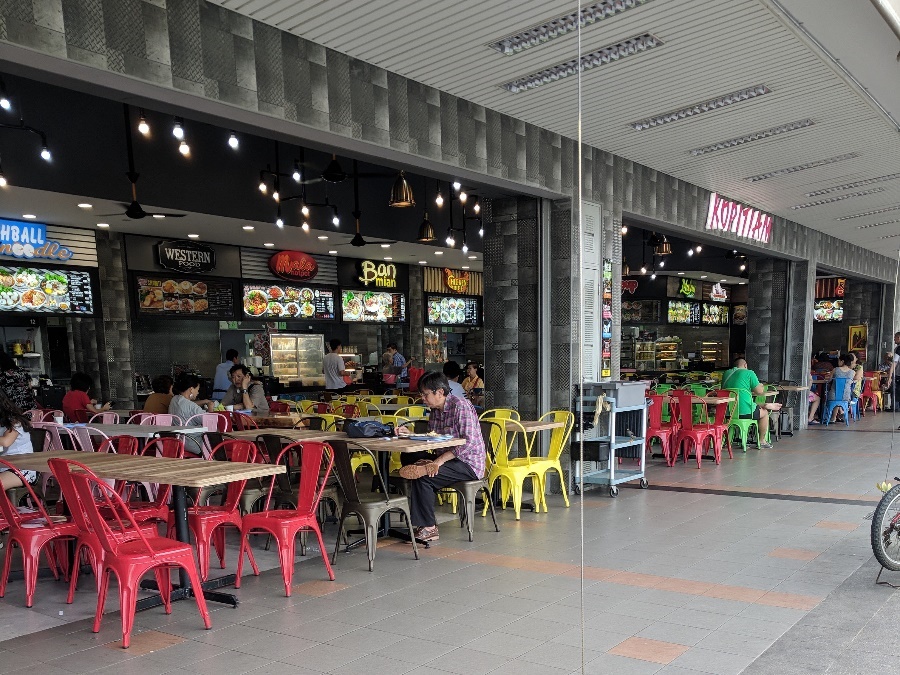


Figure A.1: Example of a Food Court: Buangkok Food Court


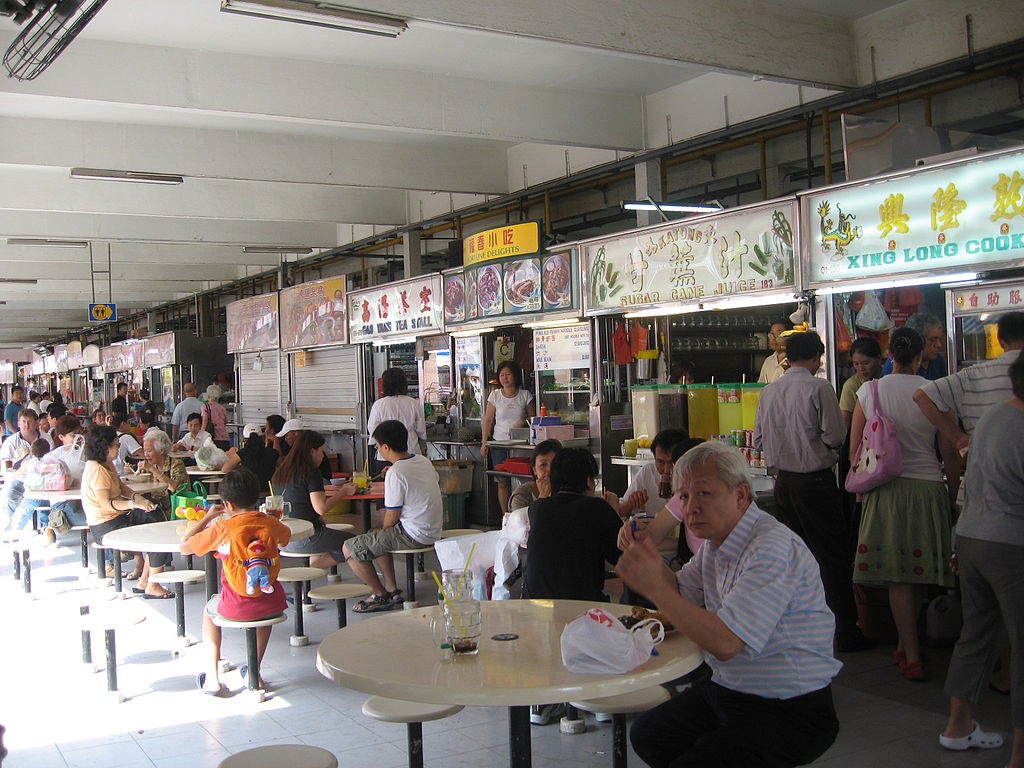


Figure A.2: Example of a Hawker Center: Marine Parade Hawker Center
