# Supplementary material for: Where we eat is who we are: a survey of food-related travel patterns to Singapore’s hawker centers, food courts and coffee shops
Source: Int J Behav Nutr Phys Act. 2020 Oct 20;17:132. doi: 10.1186/s12966-020-01031-5 (PMC7574174; doi:10.1186/s12966-020-01031-5)
Supplement: Supplementary file 2 — Additional file 2 kernel density estimations and site selection [file 12966_2020_1031_MOESM2_ESM.docx]

**Additional File 2:** **Kernel Density Estimations And Site Selection**

Information on the number of food stalls in hawker centers, which was managed by the National Environment Agency (NEA), were downloaded from an open government data portal Data.gov.sg.

Over 23,000 food outlet listings were scraped from two popular food review websites in Singapore in October 2017 and November 2017 respectively. These listings included information about the name and address of each food outlet, the food types offered, and user-generated ratings and average price of meals. These listings were merged to remove potential duplicates between and within the two datasets, by comparing the outlet names and locations to minimize double-counting. As hawker center stalls were sometimes also listed in these websites, they were identified and removed. The cleaned food listings were then combined with the NEA hawker centre dataset. The information was then further collapsed to summarize the total number of food outlets at each location point, for the entire island.

I created fixed-bandwidth kernel density estimate of food outlets throughout Singapore, using a pixel grid of 200m x 200m, and a smoothing bandwidth of sigma=200m, with edge correction. Each location is weighted by the number of food outlets located there.

I created fixed-bandwidth kernel density estimate of food outlets throughout Singapore, using a pixel grid of 200m x 200m, and a smoothing bandwidth of sigma=200m, with edge correction. Each location is weighted by the number of food outlets located there.

I developed two similar KDE maps : one for bus-stops (weighted by number of bus services at each bus-stop) and another for Mass Rapid Transit (MRT) stops. Base datasets for bus-stops and MRT stops were obtained from the Land Transport Authority’s website: data November 2017, while data on bus-services were obtained from <https://busrouter.sg/>.

Food Outlet KDE

Bus-stop KDE

MRT KDE

s

For each KDE map, I sieved out any 200mx200m pixel that did not include at least one food outlet within it, so as to focus the survey site-selection analysis on areas that had food outlets that I could station myself.

I then ranked each 200x200m pixel that had at least one food outlet within it from lowest to highest density The two ranked public transportation maps were added together, with the MRT values being weighted 10 times more than the bus-stop values to reflect the substantially higher levels of accessibility (in terms of speed of travel) offered by an MRT station compared to the bus-stop. The combined public transport map was then re-ranked from lowest to highest.

The food outlet ‘density ranked’ map and the Public Transport ‘density ranked’ map were then combined additively and rescaled from 0-100. The pixels with the highest scores (100) thus represented areas with very high density of food options as well as public transport availability. The pixels with the lowest scores (min = 0) were areas with low density of food and public transport. I then used the resultant map to identify potential high-food-high public transport, and low-food-low public transport neighborhoods where I could then recruit respondents for the survey.

To identify the low-food-high public transport, and high-food-low-public transport sites, I first inverted the ranking of the food outlet KDE map, so that pixels with high food outlet density had low scores and vice versa. This ‘inverted density ranked’ food outlet map was then combined with the Public Transport ‘density ranked’ map and rescaled to 0-100. Now, the pixels with the high scores represented areas with low density of food options but with high public transport availability. The pixels with low scores were areas with high density of food and low public transport availability. I used this map to identify potential low-food-high public transport, and high-food-low public transport neighborhoods to conduct the survey.
