# Supplementary figures and images for: Where we eat is who we are: a survey of food-related travel patterns to Singapore’s hawker centers, food courts and coffee shops
Source: Int J Behav Nutr Phys Act. 2020 Oct 20;17:132. doi: 10.1186/s12966-020-01031-5 (PMC7574174; doi:10.1186/s12966-020-01031-5)

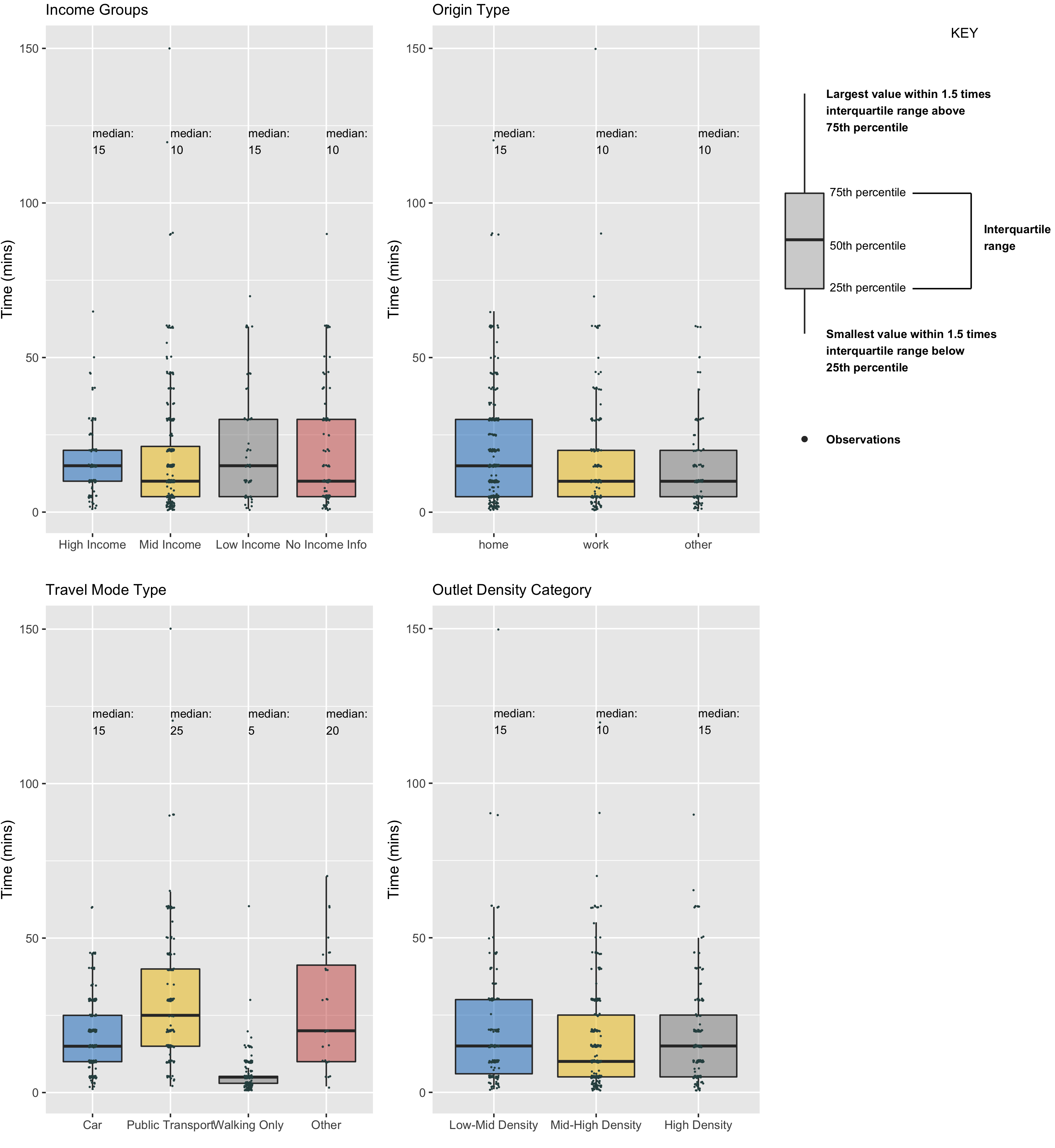

Supplement: Supplementary file 3 — Additional file 3 median travel times [file 12966_2020_1031_MOESM3_ESM.png]
